# Dysfunction of 67-kDa Laminin Receptor Disrupts BBB Integrity via Impaired Dystrophin/AQP4 Complex and p38 MAPK/VEGF Activation Following Status Epilepticus

**DOI:** 10.3389/fncel.2019.00236

**Published:** 2019-05-24

**Authors:** Hana Park, Seo-Hyeon Choi, Min-Jeong Kong, Tae-Cheon Kang

**Affiliations:** ^1^Department of Anatomy and Neurobiology, College of Medicine, Hallym University, Chuncheon, South Korea; ^2^Institute of Epilepsy Research, College of Medicine, Hallym University, Chuncheon, South Korea

**Keywords:** SMI-71, eNOS, SB202190, laminin, vasogenic edema

## Abstract

Status epilepticus (SE, a prolonged seizure activity) impairs brain-blood barrier (BBB) integrity, which results in secondary complications following SE. The non-integrin 67-kDa laminin receptor (67-kDa LR) plays a role in cell adherence to laminin (a major glycoprotein component in basement membrane), and participates laminin-mediated signaling pathways including p38 mitogen-activated protein kinase (p38 MAPK). Thus, we investigated the role of 67-kDa LR in SE-induced vasogenic edema formation in the rat piriform cortex (PC). SE diminished 67-kDa LR expression, but increased laminin expression, in endothelial cells accompanied by the reduced SMI-71 (a rat BBB barrier marker) expression. Astroglial 67-kDa LR expression was also reduced in the PC due to massive astroglial loss. 67-kDa LR neutralization led to serum extravasation in the PC concomitant with the reduced SMI-71 expression. 67-kDa LR neutralization also decreased expressions of dystrophin and aquaporin-4 (AQP4). In addition, it increased p38 MAPK phosphorylation and expressions of vascular endothelial growth factor (VEGF), laminin and endothelial nitric oxide synthase (eNOS), which were abrogated by SB202190, a p38 MAPK inhibitor. Therefore, our findings indicate that 67-kDa LR dysfunction may disrupt dystrophin-AQP4 complex, which would evoke vasogenic edema formation and subsequent laminin over-expression via activating p38 MAPK/VEGF axis.

## Introduction

Vasogenic edema is the most common type of brain edema and a result from increased capillary permeability due to breakdown in intact brain-blood barrier (BBB) that is important for the maintenance of brain homeostasis. Vasogenic edema results in an abrupt increase in intracranial pressure and abnormal blood-brain transports of serum-derived molecules, which affect neuronal excitability, properties of regenerating (recovering) vessels, white matter injury and reactive gliosis in brain tumor, exposure of insecticides, traumatic brain injury (TBI), ischemia and brain inflammation (Papadopoulos et al., 2004; Song et al., 2004; Bloch et al., 2005; Kim et al., 2010b; Li and Ehrich, 2013; Kim Y.J. et al., 2015; Ko and Kang, 2015; Bennett et al., 2016; Zhao et al., 2018; Sun et al., 2019). Therefore, the prevention or attenuation of vasogenic edema formation is one of the major therapeutic strategies, which help to alleviate undesirable secondary complications in various neurological diseases.

Status epilepticus (SE) is a medical emergency condition with significant mortality showing continuous seizure activity. SE is considered as one of the precipitating factors for temporal lobe epilepsy (TLE) that is observed in one-third of all cases of epilepsy. Human TLE patients present neuronal loss and reactive astrogliosis in various brain regions including the hippocampus, which are influenced by SE (Fountain, 2000; Koh et al., 2005; Téllez-Zenteno and Hernández-Ronquillo, 2012; Sørensen and Kokaia, 2013). Similar to human patients, SE-induced epilepsy animal models show massive neuronal loss and astroglial dysfunctions in the various brain regions. Furthermore, vasogenic edema is one of the early post-SE events in the hippocampus and extrahippocampal limbic systems including the piriform cortex (PC). Since serum extravasation during vasogenic edema formation leads to spreading depolarizations and epileptiform discharges due to neuronal hyperexcitability, dysfunction of astrocytes and potassium buffering (Sisodiya et al., 2002; Löscher and Potschka, 2005; Kim Y.J. et al., 2015; Lippmann et al., 2017), it is likely that vasogenic edema may be one of the risk factors inducing pharmacoresistant epilepsy and life-threatening complications following SE.

The BBB consists of endothelial cells, astroglial endfeet, pericytes and basement membrane (BM; Banerjee and Bhat, 2007). The BM is vital for endothelial cell adhesion, migration and stabilization under physiological conditions. The BM is a structural and specialized complex of extracellular matrix protein including laminin, fibronectin, vitronectin, collagen, and heparin sulfate proteoglycans (Tilling et al., 2002; Yurchenco and Patton, 2009). Among them, laminin is a major glycoprotein component of BMs in vessels, and is involved in cellular adhesion and migratory processes (Miner, 2008). Thus, laminin is essential for vascular development, vessel dilation and physical integrity (Thyboll et al., 2002). Interestingly, laminin expression is remarkably upregulated in the BM of vessels following SE (Biagini et al., 2008; Gualtieri et al., 2012). Indeed, we have reported that laminin over-expression is closely relevant to the repair of BBB disruption accompanied by the reconstruction of endothelial barrier following SE (Kim et al., 2014). However, the underlying mechanism of up-regulation of laminin expression in vasogenic edema formation has not been fully elucidated.

The laminin functions are mediated by cell-surface receptors of two main classes: the integrins and the non-integrin 67-kDa laminin receptor (67-kDa LR; Lesot et al., 1983; Malinoff and Wicha, 1983; Rao et al., 1983). 67-kDa LR is composed of a 32 ∼ 33 kDa precursor (approximately 37 kDa in sodium dodecyl sulfate polyacrylamide gel electrophoresis). 67-kDa LR plays a role in cell adherence to laminin and stabilizes or modulates the binding of laminin to other receptors (Pellegrini et al., 1994; Ardini et al., 1998). In addition, 67-kDa LR is involved in laminin-mediated extracellular signal-regulated kinase 1/2 (ERK1/2), c-Jun N-terminal kinase (JNK) and p38 mitogen-activated protein kinase (p38 MAPK) signaling pathways (Givant-Horwitz et al., 2004). It also binds to several exogenous agents (including prion proteins, viruses and bacteria) and regulates cellular migration, viability and cytoskeleton reorganization (Wewer et al., 1986; Rao et al., 1989; Huang and Jong, 2009; Vana et al., 2009; Kielian et al., 2010). Furthermore, 67-kDa LR is expressed in the adult rat brain (Baloui et al., 2004). With respect to these profiles of 67-kDa LR, elucidating the correlation between 67-kDa LR expression and vasogenic edema formation may be noteworthy to understand the mechanisms of BBB disruption and laminin over-expression in response to SE. Therefore, during the course of this study, we investigated the alteration in 67-kDa LR expression induced by SE, and the effect of blockade of 67-kDa LR by neutralization on laminin expression or BBB integrity in efforts to understand the role of vascular dysfunction in epileptogenic insult.

## Materials and Methods

### Experimental Animals and Chemicals

Male Sprague-Dawley (SD) rats (7 weeks old) were used in the present study. Animals were housed in a controlled temperature (22 ± 2°C), humidity (55 ± 5%) and a light-dark cycle (12:12). Food and tap water were provided *ad libitum* throughout the experiments. All experimental protocols described below were approved by the Institutional Animal Care and Use Committee of Hallym University (Chuncheon, South Korea). Every effort was made to reduce the number of animals employed and to minimize animal’s discomfort. All reagents were obtained from Sigma-Aldrich (St. Louis, MO, United States), except as noted.

### SE Induction

Rats were pretreated with an intraperitoneal injection of LiCl (127 mg/kg i.p) 24 h before the pilocarpine (PILO) treatment. Animals were intraperitoneally (i.p) treated with PILO (30 mg/kg) 20 min after atropine methylbromide (5 mg/kg i.p.). PILO injection resulted in stereotypical behavioral responses, which included the following: akinesia, staring, salivation, facial automatisms, slight tremors and head bobbing. These behavior responses built up progressively into motor limbic seizures that recurred repeatedly and rapidly developed into SE characterized by forelimb clonus and tonic-clonic seizures with loss of righting reflexes. SE was defined by continuous or intermittent seizures without full recovery between seizures. Control animals received an equal volume of normal saline instead of PILO after the pretreatment with atropine methylbromide. Diazepam (Valium; Hoffman la Roche; 10 mg/kg, i.p.) was administered 2 h after onset of SE and repeated, as needed. Three days after SE, animals were used for Western blot and immunohistochemistry.

### Surgery

Under Isoflurane anesthesia (3% induction, 1.5–2% for surgery and 1.5% maintenance in a 65:35 mixture of N_2_O:O_2_), animals were infused each chemical into the right lateral ventricle (1 mm posterior; 1.5 mm lateral; -3.5 mm depth to the bregma) with a brain infusion kit 1 and an Alzet 1003D osmotic pump (Alzet, United States) for 3 days. Osmotic pump contained (1) control IgG (Abcam, #ab37425, United Kingdom, 50 ug/ml) + vehicle, (2) control IgG + SB202190 (a p38 MAPK inhibitor, 0.3 mg/ml), (3) anti-67-kDa LR IgG (Abcam, #133645, United Kingdom, 50 ug/ml) + vehicle and (4) anti-67-kDa LR IgG (Abcam, #133645, United Kingdom, 50 ug/ml) + SB202190 (0.3 mg/ml). In pilot study and our previous studies (Kim et al., 2016; Ko and Kang, 2017), each compound treatment did not show behavioral and neurological defects and could not change the seizure susceptibility and seizure severity in response to PILO in normal animals. Three days after surgery (infusion), animals were used for Western blot and immunohistochemistry.

### Western Blot

After animals were sacrificed via decapitation, the PC was obtained. The PC tissues were homogenized, and determined protein concentration using a Micro BCA Protein Assay Kit (Pierce Chemical, United States). Western blot was performed by the standard protocol. Membranes were incubated with primary antibody against 67-kDa LR (Abcam, #133645, United Kingdom, diluted 1:1,000), aquaporin-4 (AQP4, Alomone labs, #AQP-004, Israel, 1:5,000), dystrophin (Abcam, #ab15277, United Kingdom, diluted 1:5,000), endothelial nitric oxide synthase (eNOS, Abcam, #ab66127, United Kingdom, diluted 1:1,000), laminin (Abcam, #ab11575, United Kingdom, diluted 1:1,000), p38 MAPK (Cell signaling, #9212, United States, diluted 1:1000) or p-p38 MAPK (Abbiotec, #251246, United States, diluted 1:200), rat IgG (Vector, #PI9400, United States, diluted 1:200) or VEGF (Abcam, #ab46154, United Kingdom, diluted 1:1000) and visualized by an ECL Kit (Amersham, United States). The bands were detected and quantified on ImageQuant LAS4000 system (GE Healthcare, United States). As an internal reference, rabbit anti-β-actin primary antibody (#A5316, 1:5000) was used. The values of each sample were normalized with the corresponding amount of β-actin. The ratio of phosphoprotein to total protein was described as phosphorylation ratio.

### Immunohistochemistry

Rats were anesthetized with urethane anesthesia (1.5 g/kg, i.p.) and intracardially perfused with 4% paraformaldehyde in 0.1 M phosphate buffer (PB, pH 7.4). Subsequently, brains were post-fixed in the same fixative overnight, followed by cryoprotection with 30% sucrose/0.1 M PBS, and sectioned at 30 μm with a cryo-microtome. Standard procedures for immunohistochemistry were used to detect vasogenic edema formation. Briefly, free-floating sections were washed three times in PBS (0.1 M, pH 7.3). Next, to inactivate the endogenous peroxidase, slides were incubated in 3% H_2_O_2_ and 10% methanol in PBS (0.1 M) for 20 min at room temperature. Later, sections were incubated in biotinylated rat IgG (Vector, #BA9400, United States, diluted 1:200) and ABC complex (Vector, #PK-6100, United States, diluted 1:200). Tissue sections were developed in 3,3′-diaminobenzidine in 0.1 M Tris buffer and mounted on gelatin-coated slides. The volume of vasogenic edema lesion in the PC was measured by AxioVision Rel. 4.8 software and estimated by the modified Cavalieri method: *V* = Σ*area* × section thickness (30 μm) × 1/the fraction of the sections (1/6). The volumes are reported in mm^3^ (Kim et al., 2016).

Some sections were incubated with a cocktail solution containing the primary antibodies against 67-kDa LR (Abcam, #ab133775, United Kingdom, diluted 1:500), AQP4 (Alomone labs, #AQP-004, Israel, diluted 1:500), dystrophin (Abcam, #ab15277, United Kingdom, diluted 1:500), eNOS (Abcam, #ab66127, United Kingdom, diluted 1:500), glial fibrillary acidic protein (GFAP, Millipore, #MAB3402, United States, diluted 1:5,000), laminin (Abcam, #ab11575, United Kingdom, diluted 1:200) and SMI-71 (Covance, #SMI-71R, United States, diluted 1:1000) in PBS containing 0.3% Triton X-100 overnight at room temperature. Thereafter, sections were visualized with appropriate Cy2- and Cy3-conjugated secondary antibodies. Immunoreaction was observed using an Axio Scope microscope (Carl Zeiss Inc., Germany). To establish the specificity of the immunostaining, a negative control test was carried out with preimmune serum instead of the primary antibody. No immunoreactivity was observed for the negative control in any structures (data not shown). All experimental procedures in this study were performed under the same conditions and in parallel. To quantify relative fluorescence intensity, sections (10 sections per each animal) were captured using an AxioImage M2 microscope. A 300-μm^2^ box was then randomly placed within the region of interest. Thereafter, mean fluorescence intensity of each section was measured by using AxioVision Rel. 4.8 software. Fluorescence intensity measurements were represented as the number of a 256 gray scale. Fluorescence intensity values were corrected by subtracting the average values of background noise (mean background intensity) obtained from five image inputs. Fluorescent intensity of each section was standardized by setting the threshold level (mean background intensity obtained from five image inputs). Manipulation of the images was restricted to threshold and brightness adjustments to the whole image (Kim et al., 2013, 2014; Kim and Kang, 2017).

### Statistical Analysis

Quantitative data are expressed as mean ± standard error of the mean. Data are analyzed by Student *t*-test or one-way ANOVA followed by Newman–Keuls *post hoc* test. A *p* < 0.05 is considered to be statistically different.

## Results

### SE-Induced Reduction in 67-kDa LR Expression in Vasogenic Edema Lesion

**Figure 1:**
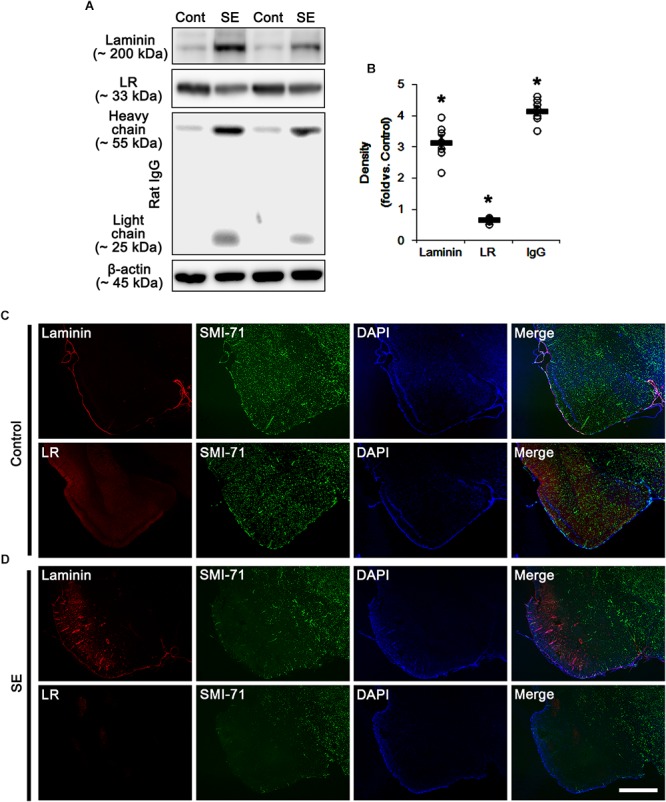
Alterations in laminin and 67-kDa LR (LR) expression in the PC following SE. **(A)** Western blot image for expression levels of laminin and 67-kDa LR, and serum extravasation following SE. SE increases laminin expression and serum extravasation, but reduces 67-kDa LR expression. **(B)** Quantitative values (mean ± S.E.M) of the Western blot data concerning expression levels of laminin and 67-kDa LR, serum extravasation induced by SE (*n* = 7, respectively). Open circles indicate each individual value. Horizontal bars indicate mean value. Significant differences are ^∗^*p* < 0.05 vs. control animals (Student *t*-test). **(C,D)** Representative photographs of expression levels of laminin and 67-kDa LR in vasogenic edema (SMI-71-deleted) lesion in the PC in control **(C)**- and post-SE condition **(D)**. In SMI-71-deleted area, laminin expression is enhanced, while 67-kDa LR expression is decreased. Bar = 400 μm.

Consistent with our previous studies (Sheen et al., 2011; Kim et al., 2012, 2013, 2014), Figure 1 shows that SE led to serum extravasation in the PC (*p* < 0.05 vs. control animals, Student *t*-test, *n* = 7, respectively). In addition, SE increased laminin expression to 3.2-fold of control level in the PC (*p* < 0.05 vs. control animals, Student *t*-test, *n* = 7, respectively; Figure 1A,B and Supplementary Figure S1). However, 67-kDa LR expression level was diminished to ∼ 0.68-fold of control level in this region (*p* < 0.05 vs. control animals, Student *t*-test, *n* = 7, respectively; Figure 1A,B and Supplementary Figure S1). Furthermore, SE reduced SMI-71 (a rat BBB barrier antibody) expression in the PC (Figure 1C,D), which is an indicative of vasogenic edema formation (Sheen et al., 2011; Kim et al., 2012, 2013, 2014). However, laminin expression was increased in SMI-71-deleted areas in the PC (Figure 1C,D). Although laminin over-expression would predict astroglial loss following SE (Gualtieri et al., 2012; Willis et al., 2013), our previous study has revealed that laminin over-expression is not relevant to astroglial death induced by SE. This is because laminin expression is unaltered in the frontoparietal cortex where vasogenic edema is not detected following SE. Furthermore, laminin over-expression is ameliorated by inhibition of vasogenic edema formation and during recovery of vasogenic edema (Kim et al., 2014). Thus, the present data revel that SE-induced BBB disruption may be a cause of laminin over-expression, and that the subsequent laminin over-expression may be one of compensatory responses for recovery of BBB integrity. In contrast to laminin expression, 67-kDa LR expression was decreased in SMI-71-deleted areas in the PC (Figure 1C,D), indicating 67-kDa LR expression may be relevant to BBB integrity.

### Decreased 67-kDa LR Expression in Astrocytes Following SE

**Figure 2:**
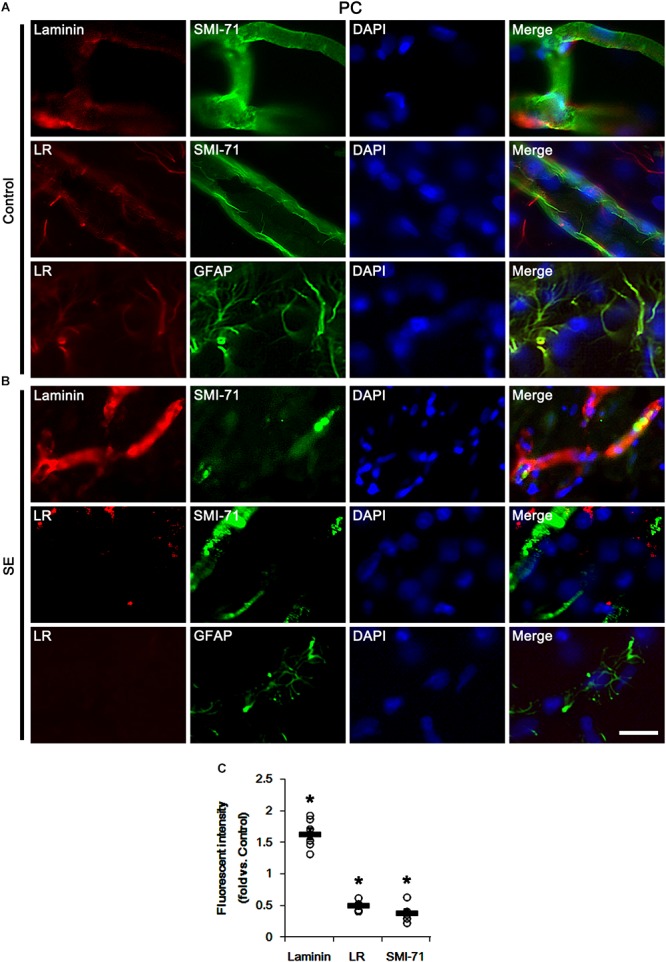
Alterations in laminin and 67-kDa LR (LR) expression in capillaries and astrocytes within the PC following SE. **(A,B)** Representative photographs of SMI-71, laminin and 67-kDa LR expression in control **(A)**- and post-SE animals **(B)**. SE increases laminin expression, but reduces 67-kDa LR expression, accompanied by BBB disruptions (SMI-71 degradation) in capillaries. SE also diminished astroglial 67-kDa LR expression. Bar = 12.5 μm. **(C)** Quantitative values (mean ± S.E.M) of the fluorescent intensities of laminin, 67-kDa LR and SMI-71 (*n* = 7, respectively). Open circles indicate each individual value. Horizontal bars indicate mean value. Significant differences are ^∗^*p* < 0.05 vs. control animals (Student *t*-test).

Next, we investigated the cellular localizations of laminin and 67-kDa LR in the PC. In control animals, laminin expression was detected in endothelial cells, and 67-kDa LR expression was weakly observed in endothelial cells and astrocytes within the PC (Figure 2A). Following SE, laminin expression was increased in vasogenic edema lesion within the PC where astroglial loss was observed (*p* < 0.05 vs. control animals, Student *t*-test, *n* = 7, respectively; Figure 2B,C). In contrast to laminin, 67-kDa LR expression was diminished in endothelial cells accompanied by loss of SMI-71 expression. Astroglial 67-kDa LR expression was also reduced in the PC due to massive astroglial loss. In addition, the remaining astrocytes in this region showed the decrease in 67-kDa LR expression (*p* < 0.05 vs. control animals, Student *t*-test, *n* = 7, respectively; Figure 2B,C). These findings indicate that SE-induced down-regulation of 67-kDa LR expression may be relevant to vasogenic edema formation.

### Laminin Over-Expression and BBB Disruption Induced by 67-kDa LR Neutralization

**Figure 3:**
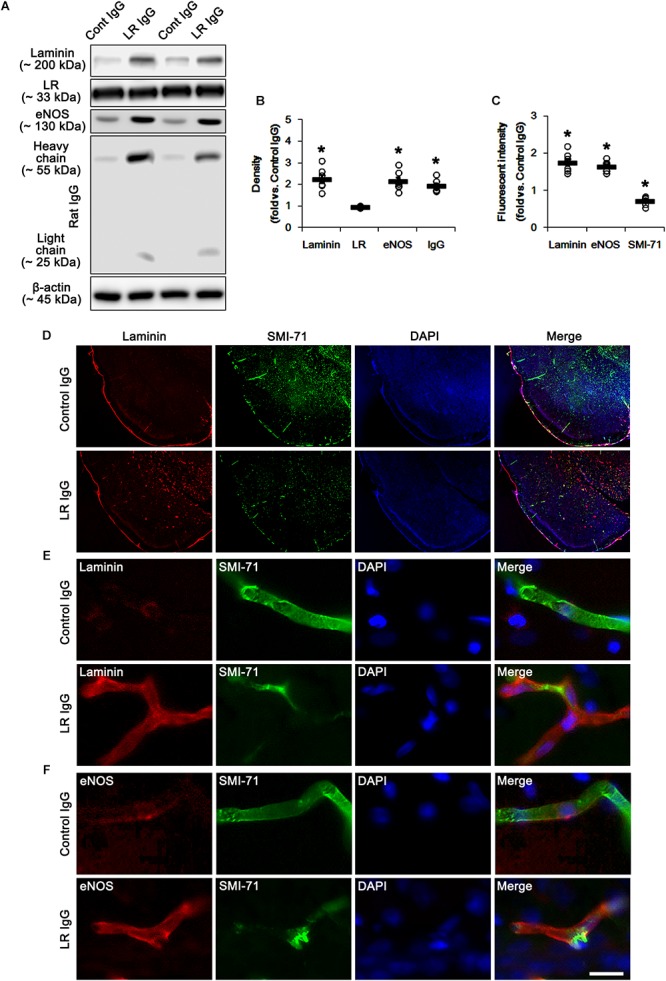
67-kDa LR (LR) neutralization-induced serum extravasation in normal animals. **(A)** Western blot image for expression levels of laminin, 67-kDa LR and eNOS, and serum extravasation induced by 67-kDa LR neutralization. 67-kDa LR IgG infusion increases expression levels of laminin and eNOS and serum extravasation, without changing 67-kDa LR expression. **(B)** Quantitative values (mean ± S.E.M) of the Western blot data concerning expression levels of laminin, 67-kDa LR and eNOS, and serum extravasation induced by 67-kDa LR neutralization (*n* = 7, respectively). Open circles indicate each individual value. Horizontal bars indicate mean value. Significant differences are ^∗^*p* < 0.05 vs. control IgG (Student *t*-test). **(C)** Quantitative values (mean ± S.E.M) of the fluorescent intensities of SMI-71, eNOS and laminin (*n* = 7, respectively). Significant differences are ^∗^*p* < 0.05 vs. control IgG (Student *t*-test). **(D–F)** Representative photographs of expression levels of laminin, eNOS, and SMI-71 in the PC. Bar = 400 μm **(D)** and 12.5 μm **(E,F)**.

In our previous study, we have reported that control IgG and interleukin-18 antibody infusions differently affects SE-induced vasogenic formation in the PC (Jung et al., 2012). To directly confirm the role of 67-kDa LR in vasogenic edema formation, thus, we applied 67-kDa LR antibody infusion (neutralization) to control animals and explored its effect on BBB integrity. As compared to control IgG, 67-kDa LR neutralization led to serum extravasation in the PC without changing 67-kDa LR expression level (*p* < 0.05 vs. control IgG, Student *t*-test, *n* = 7, respectively; Figure 3A,B and Supplementary Figure S1). 67-kDa LR antiserum increased laminin expression to 2.26-fold of control IgG level in the PC (*p* < 0.05 vs. control IgG, Student *t*-test, *n* = 7, respectively; Figure 3A,B and Supplementary Figure S1). It also up-regulated laminin and eNOS expressions in endothelial cells, accompanied by the reduced SMI-71 expression in this region (*p* < 0.05 vs. control IgG, Student *t*-test, *n* = 7, respectively; Figure 3A–F). Together with the data obtained from SE induction, our findings indicate that the blockade of 67-kDa LR functionality may lead to vasogenic edema formation by BBB breakdowns, and that 67-kDa LR may negatively regulate laminin expression in BM of brain capillary.

### 67-kDa LR Neutralization-Induced Reductions in Dystrophin and AQP4 Expression in Astrocytes and Endothelial Cells

**Figure 4:**
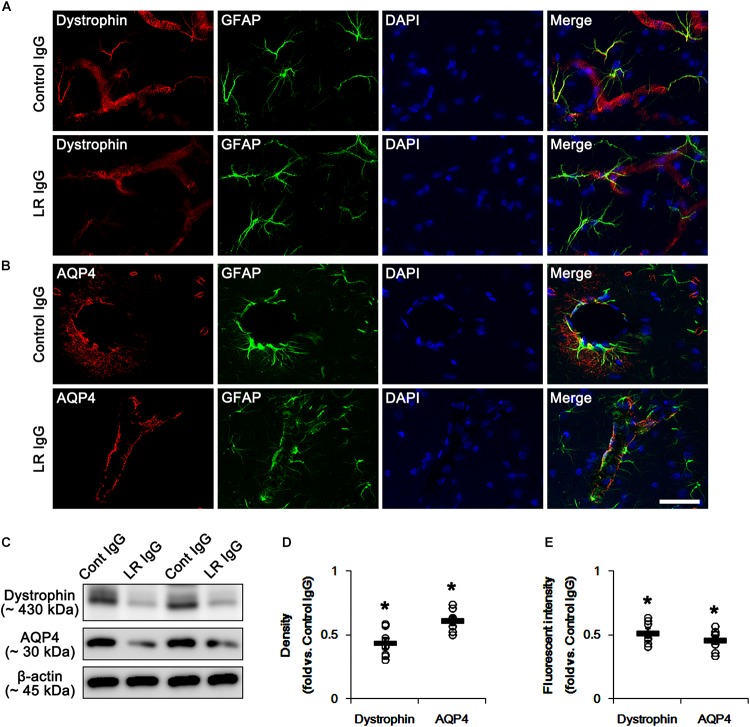
Effect of 67-kDa LR (LR) neutralization on dystrophin and AQP4 expression in the PC of normal animals. **(A,B)** Representative photographs of dystrophin **(A)** and AQP4 **(B)** expressions in the PC. As compare to vehicle, 67-kDa LR neutralization leads to the reduction in dystrophin and AQP4 expressions in the PC. Bar = 12.5 μm. **(C)** Western blot image for expression levels of dystrophin and AQP4. **(D,E)** Quantitative values (mean ± S.E.M) of the Western blot data **(D)** and the fluorescent intensities concerning expression levels of dystrophin and AQP4 induced by 67-kDa LR neutralization (*n* = 7, respectively). Open circles indicate each individual value. Horizontal bars indicate mean value. Significant differences are ^∗^*p* < 0.05 vs. control IgG (Student *t*-test).

In the present study, we found that SE and 67-kDa LR neutralization induced laminin over-expression, which is closely relevant to the repair of BBB disruption accompanied by the reconstruction of endothelial barrier (Kim et al., 2014). Interestingly, laminin interacts with dystrophin-glycoprotein complex that maintains the anchoring astroglial endfeet to BM, the polarized expression of AQP4 water channel and the BBB integrity (Neely et al., 2001; Nico et al., 2003; Wolburg et al., 2009), which are down-regulated by SE (Kim et al., 2010b; Sheen et al., 2011). With respect to these previous reports, it is likely that 67-kDa LR-mediated regulation of dystrophin expression would maintain AQP4 expression and BBB integrity under physiological condition. Consistent with our previous studies (Kim et al., 2010b; Sheen et al., 2011), the expression of dystrophin and AQP4 were observed in astrocytes and brain capillaries in control IgG-infused animals. 67-kDa LR neutralization decreased dystrophin and AQP4 expressions without astroglial loss in the PC (*p* < 0.05 vs. control IgG, Student *t*-test, *n* = 7, respectively; Figure 4A–E and Supplementary Figure S2). Therefore, our findings indicate that 67-kDa LR may play a role in maintenance of dystrophin-AQP4 complex in astrocytes and brain capillaries.

### Elevated p38 MAPK Phosphorylation Induced by 67-kDa LR Neutralization

The remaining question is what underlying signaling pathway is involved in vasogenic edema formation induced by 67-kDa LR neutralization. Recently, we have reported that p38 MAPK-mediated VEGF over-expression leads to BBB breakdown following SE (Kim et al., 2016; Kim and Kang, 2017). Interestingly, reduced 67-kDa LR expression increased p38 MAPK phosphorylation (activation), regardless of the exposure to exogenous laminin (Givant-Horwitz et al., 2004). Furthermore, mice lacking dystrophin show but a high endogenous p38 MAPK phosphorylation, as compared to wild-type animals (Smythe and Forwood, 2012; Wissing et al., 2014). Therefore, it is likely that 67-kDa LR-mediated regulation of dystrophin expression may be one of the up-stream signals to regulate BBB integrity via p38 MAPK/VEGF axis.

**Figure 5:**
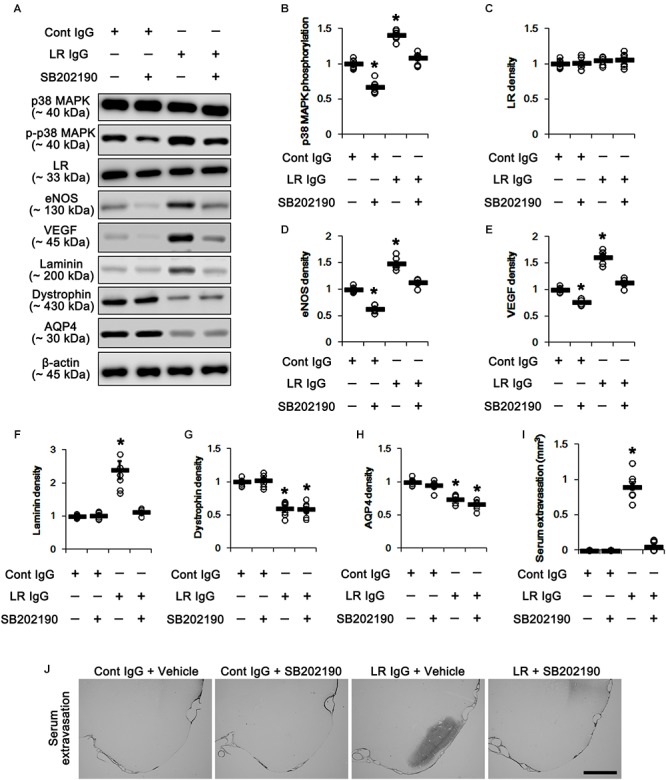
Effect of 67-kDa LR (LR) neutralization and SB202190 co-treatment on serum extravasation in the PC of normal animals. **(A)** Western blot image for expression levels of p38 MAPK, phospho (p)-p38 MAPK, 67-kDa LR, eNOS, VEGF, laminin, dystrophin, and AQP4. **(B–H)** Quantitative values (mean ± S.E.M) of the Western blot data concerning expression levels of phospho (p)-p38 MAPK, 67-kDa LR, eNOS, VEGF, laminin, dystrophin, and AQP4 (*n* = 7, respectively). Open circles indicate each individual value. Horizontal bars indicate mean value. Significant differences are ^∗^*p* < 0.05 vs. control IgG (one-way ANOVA). **(I,J)** Quantitative values (mean ± S.E.M) of areas (*n* = 7, respectively; **(I)** and representative photographs **(J)** of serum extravasation in the PC. ^∗^*p* < 0.05 vs. control IgG (one-way ANOVA). Bar = 400 μm.

In the present study, 67-kDa LR neutralization led to up-regulation of p38 MAPK phosphorylation and VEGF expression in the PC, as compared to control IgG (*p* < 0.05 vs. control IgG, one-way ANOVA, *n* = 7, respectively; Figure 5A,B,E and Supplementary Figure S2). SB202190 (a p38 MAPK inhibitor) co-treatment did not affect 67-kDa LR expression levels in both control IgG- and 67-kDa LR IgG-infused animals (Figure 5A,C and Supplementary Figure S2). However, SB202190 co-treatment abrogated p38 MAPK activation, eNOS induction, VEGF induction and laminin over-expression induced by 67-kDa LR neutralization, although it could not abolish reductions in dystrophin and AQP4 expressions (*p* < 0.05 vs. vehicle, one-way ANOVA, *n* = 7, respectively; Figure 5A–H and Supplementary Figure S2). Furthermore, SB202190 inhibited serum extravasation in the PC induced by 67-kDa LR IgG (*p* < 0.05 vs. vehicle, one-way ANOVA, *n* = 7, respectively; Figure 5I,J). Thus, our findings indicate that the disruption of dystrophin-AQP4 complex by 67-kDa LR dysfunctions may trigger vasogenic edema formation and laminin over-expression by activating p38 MAPK-mediated VEGF expression.

## Discussion

**Figure 6:**
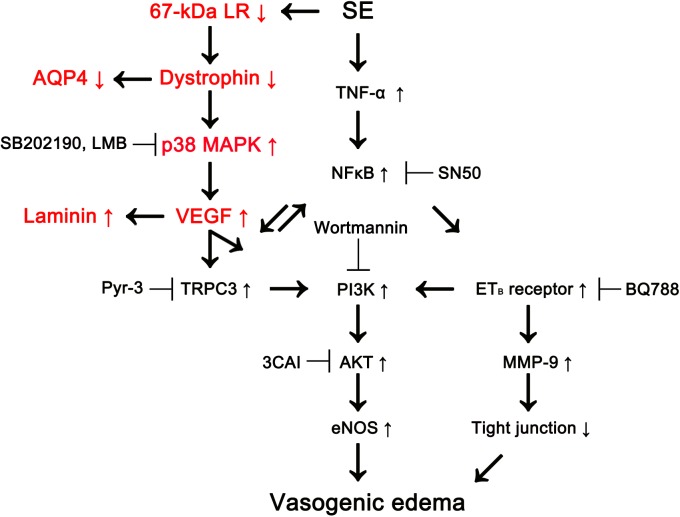
Scheme of role of 67-kDa LR in vasogenic edema formation induced by SE based on the present data and previous reports (Kim et al., 2013, 2016; Kim J.Y. et al., 2015; Min and Kang, 2016). SE reduces 67-kDa LR and dystrophin expressions in astrocytes and endothelial cells, which activates p38 MAPK/VEGF axis. Subsequently, VEGF increases eNOS activity via transient receptor potential canonical channel-3 (TRPC3)/PI3K/AKT signaling pathway, which evokes vasogenic edema formation, along with ET_B_ receptor activation. Down-regulation of dystrophin also decreases AQP4 expression leading to exacerbation of vasogenic edema. In addition, VEGF induces laminin over-expression for recovery of damaged brain capillaries.

Recently, we have reported that p38 MAPK-mediated VEGF over-expression leads to phosphatidylinositol-3-kinase (PI3K)/AKT-mediated eNOS activation during SE-induced vasogenic edema formation, which is also regulated by tumor necrosis factor-α (TNF-α)/nuclear factor-κB (NF-κB)/endothelin B (ET_B_) receptor-mediated signaling pathway (Kim et al., 2016; Min and Kang, 2016; Kim and Kang, 2017). However, we could not elucidate the up-stream effectors/signaling pathways concerning p38 MAPK/VEGF-mediated vasogenic edema formation. In the present study, we found that 67-kDa LR might inhibit this signaling pathway by maintaining the integrity of dystrophin-AQP4 complex (Figure 6).

Consistent with previous studies (Castronovo et al., 1991; Guo et al., 1992), the present study shows that 67-kDa LR is identified in astrocytes and vascular endothelial cells. Furthermore, SE diminished its expression level in the PC. The present data also reveal that 67-kDa LR neutralization evoked the up-regulations of p38 MAPK phosphorylation, VEGF induction and eNOS expression in the normal animals, accompanied by serum extravasation and the reduced dystrophin expression, which were abrogated by SB202190. VEGF is a chemokine inducing impairment of BBB integrity, which potentially causes vasogenic edema (Pavlicek et al., 2000; Walter et al., 2001; Argaw et al., 2009; Bates, 2010; Kim et al., 2016; Kim and Kang, 2017). Aforementioned, 67-kDa LR and dystrophin expression levels regulate p38 MAPK activity (Givant-Horwitz et al., 2004; Smythe and Forwood, 2012; Wissing et al., 2014), and activation of p38 MAPK-VEGF axis participates in SE-induced vasogenic edema via the increased PI3K/AKT-mediated eNOS expression (Kim et al., 2016; Kim and Kang, 2017). Thus, our findings indicate that the reduced 67-kDa LR expression may be one of the up-stream molecules to regulate this signaling pathway during vasogenic edema formation. On the other hand, it is plausible that 67-kDa LR neutralization-induced up-regulations of p38 MAPK phosphorylation, VEGF induction, laminin and eNOS expression would be an adaptive reaction to rescue the BBB breakdown rather than a part of further detrimental mechanisms. If this hypothesis were true, the blockade of p38 MAPK signaling pathway would deteriorate vasogenic edema formation induced by 67-kDa LR neutralization. However, the present study reveals that SB202190 co-treatment mitigated serum extravasation induced by 67-kDa LR IgG infusion. Therefore, our findings suggest that 67-kDa LR may negatively regulate p38 MAPK/VEGF axis, which evokes BBB disruption.

In addition to VEGF, p38 MAPK activates cytosolic phospholipase A2 (cPLA2) that mediates arachidonic acid metabolism causing BBB dysfunction and edema. Indeed, p38 MAPK/cPLA2 pathway promotes BBB disruption with secondary vasogenic edema after ischemia-reperfusion injury (Nito et al., 2008). Furthermore, p38 MAPK activates Src family kinase regulates BBB permeability in response to VEGF. p38MAPK/VEGF/Src kinase pathway is also involved in BBB disruption and vasogenic edema formation via inflammation-associated BBB dysfunction in subarchnoid hemorrhage (Han et al., 2018), ischemic stroke (Liang et al., 2009) and cold-induced brain injury, but not experimental pneumococcal meningitis (Paul et al., 2007). Therefore, it is not excluded the possibilities that p38 MAPK-VEGF axis would lead to SE-induced vasogenic edema formation activating the cPLA2 and Src pathways.

Brain-blood barrier breakdown induces the leakage of albumin from blood into brain tissue. Albumin activates microglia and affects its cytokine releases and synthesis of inducible nitric oxide synthase (iNOS) via p38 MAPK pathway (Ralay Ranaivo and Wainwright, 2010; Yang et al., 2016). In addition, albumin results in the productions of inflammatory mediators, matrix metalloproteinase-9 (MMP-9) and myosin light chain kinase (MLCK) in astrocytes through p38 MAPK activation, which lead to disruption of vascular barrier integrity by the reorganization of the cytoskeleton and the dissociation of laminin as well as tight junctions (Rossi et al., 2011; Ralay Ranaivo et al., 2012; Kim Y.J. et al., 2015; Amtul et al., 2018). With respect to these previous reports, it is likely that extravasation of albumin may also reinforce p38 MAPK/VEGF signal pathway, accompanied by down-regulation of 67-kDa LR expression, following vasogenic edema formation.

The interaction between laminin and dystrophin plays an important role in the BBB integrity and AQP4 polarization in brain capillaries and astrocytes (Neely et al., 2001; Nico et al., 2003; Wolburg et al., 2009; Kim et al., 2010b; Sheen et al., 2011). Absence of dystrophin or deletion of base pairs in the dystrophin gene is a cause of Duchenne and Becker muscular dystrophy, which are allelic X-linked disorders with a progressive muscle weakness, a static cognitive impairment, autism and problems of behavior and attention (Young et al., 2008; de Brouwer et al., 2014). In mice models, deletion of dystrophin increases blood-retinal barrier (BRB) permeability by VEGF over-expression, and AQP4 delocalization/down-regulation (El Mathari et al., 2015; Vacca et al., 2016). In the brain, dystrophin deletion also reduces AQP4 expression over perivascular astroglial endfoot membranes (Enger et al., 2012). However, the regulatory signaling pathways for AQP4 expression have been still controversial. For example, p38 MAPK and JNK inhibitors decrease AQP4 protein levels in cultured human primary cortical astrocytes (Salman et al., 2017). In contrast, temozolomide (an effective drug for malignant glioma) decreases AQP4 expression level with increasing p38 MAPK phosphorylation, which is blocked by p38 MAPK inhibitor (Chen et al., 2017). Similarly, ERK1/2 pathway down-regulates AQP4 expression in scratch-injured astrocytes (Shi et al., 2015), while ERK1/2 activation up-regulates AQP4 expression in oxygen-glucose deprivation-recovery-induced injury (Qi et al., 2011). In the present study, 67-kDa LR neutralization diminished expression levels of dystrophin and AQP4 in the PC. These findings indicate that 67-kDa LR-mediated regulation of dystrophin expression may maintain AQP4 expression and BBB integrity under physiological condition. The present study also demonstrates that SB201290 did not affect expression levels of dystrophin and AQP4 in control IgG-infused animals. Furthermore, SB202190 co-treatment could not affect decreases in dystrophin and AQP4 expressions, although it abolished serum extravasation induced by 67-kDa LR neutralization. These findings indicate that impairment of dystrophin-AQP4 complex without activation of p38 MAPK/VEGF signaling pathway may not trigger BBB disruption in the PC, and that p38 MAPK activity may not influence expressions of dystrophin and AQP4. Indeed, AQP4 deletion does not affect the brain morphology and baseline intracranial pressure under physiological condition, while it worsens vasogenic edema in a freeze-injury model and a brain tumor edema model (Papadopoulos et al., 2004). Furthermore, acetazolamide (an AQP4 inhibitor) exacerbates vasogenic edema and astroglial loss in the PC following SE, although it did not induce vasogenic edema under physiological conditions (Kim et al., 2010b). Therefore, our findings suggest that the disruption of dystrophin may trigger BBB breakdown by activating p38 MAPK-mediated VEGF expression, and subsequently may aggravate vasogenic edema due to diminishing AQP4-dependent vasogenic water elimination.

Laminin is a key component of vascular BMs and contributes to vascular permeability by generating the physical barrier of the gliovascular BM and BBB maturation (Menezes et al., 2014). Laminin is also involved in the communication among the cellular components forming the gliovascular units via the integrins and non-integrin receptors (Lesot et al., 1983; Malinoff and Wicha, 1983; Rao et al., 1983; Menezes et al., 2014). In the present study, SE resulted in the down-regulated 67-kDa LR expression accompanied by laminin over-expression. Furthermore, 67-kDa LR neutralization evoked the similar effects in normal animals. Considering the essential roles of laminin in brain vascular development and vessel integrity (Thyboll et al., 2002) and its proteolytic cleavage by 67-kDa LR (Ardini et al., 2002), it is likely that the SE-induced reduction in 67-kDa LR expression and 67-kDa LR neutralization may up-regulate laminin expression to inhibit or recover vasogenic edema formation. Furthermore, SB202190 co-treatment abrogated laminin expression and serum extravasation induced by 67-kDa LR neutralization. Therefore, our findings indicate that 67-kDa LR dysfunction may up-regulate laminin expression via p38 MAPK/VEGF signaling pathway, and that laminin over-expression may be a secondary adaptive response to serum extravasation.

Similar to 67-kDa LR neutralization, recombinant tissue plasminogen activator (r-tPA) induces BBB disruption, and subsequently results in vasogenic edema (Copin et al., 2011), which is mitigated by p38 MAPK inhibitor (Garraud et al., 2016). Rosuvastatin (a lipid-lowering agent) also ameliorates r-tPA-induced BBB damage by reducing the activities of JNK and p38 MAPK (Lu et al., 2018). Indeed, tPA activates ERK1/2, JNK, AKT and p38 MAPK signaling pathways (Pineda et al., 2012). Thus, it is plausible that 67-kDa LR neutralization may lead to vasogenic edema through tPA activation and various signaling pathways as well as p38 MAPK. Further studies are needed to validate the relationship between 67-kDa LR and tPA and the related signaling pathways.

Pilocarpine is a cholinergic agent, which activates muscarinic M1 receptors (Hamilton et al., 1997; Kim and Kang, 2011). Acetylcholine induces vasodilation by eNOS activation (Gericke et al., 2013) and cholinesterase inhibitors such as chlorpyrifos and paraoxon (organophosphorus insecticides) evoke vasogenic edema (Song et al., 2004; Li and Ehrich, 2013). Thus, it is presumable that the direct PILO-mediated eNOS activation would affect seizure activity and vasogenic edema formation, although we blocked its peripheral cholinergic effects by atropine methylbromide in the present study. Recently, our laboratory reveals that *N*-nitro-L-arginine methyl ester (L-NAME, a non-selective NOS inhibitor) abrogates NO synthesis without altering seizure activity in response to PILO by the real-time simultaneous monitoring of NO and EEG. Thus, we have speculated that NO synthesis may be a consequent response to seizure activity, and that NO itself may not be directly involved in ictogenesis (Lee and Kim, 2018; Jeon and Kim, 2018). Furthermore, ET_B_ receptor antagonist attenuated vasogenic edema formation via reducing eNOS expression without altering PILO-induced seizure activity (Kim J.Y. et al., 2015). Therefore, it is unlikely that the direct cholinergic effect of PILO on eNOS activity may not influence seizure activity and vasogenic edema formation, at least in our model.

Various brain insults induce an osmotic potential across central necrotic tissue, inflammatory reaction, tight junction degradation and protein extravasation, which are regulated by inflammatory cytokines, MMP, VEGF and leukocyte/neutrophil infiltration (for review; Jha et al., 2019). Similar to other disease models, SE-induced vasogenic edema formation is relevant to neuroinflammation, MMP-9 activation and neutrophil infiltration (Kim et al., 2010a, 2012; Jung et al., 2012; Kim J.Y. et al., 2015). Thus, it is likely that the fundamental mechanisms of vasogenic edema formation induced by SE may be similar to those under various pathophysiological conditions. However, there are many underlying factors including age, sex and genetics to contribute vasogenic edema formation in animal model and human patients. For example, the major population of patients of posterior reversible encephalopathy syndrome (PRES), which is a neurotoxic encephalopathic state showing seizures and SE with white matter vasogenic edema in occipital and parietal lobes, is woman (Kamiya-Matsuoka and Tummala, 2017). Therefore, it seems that vasogenic edema would show gender-dependent characteristics. Indeed, sex steroids influences vascular reactivity in the brain via various signaling molecules including eNOS (Krause et al., 2006; Parikh et al., 2017). However, AQP4 expression is down-regulated in glioma patients without gender-dependent difference in AQP4 expression, although sex hormones such as estrogen, progesterone and testosterone modulate AQP4 expression in astrocytes (Warth et al., 2007). Ischemic stroke also shows the relative protection from brain injury in the female as compared with male, which is conferred by endogenous sex steroids. However, AQP4 is not involved in gender-specific differences in stroke volume and finally, the perivascular pool of AQP4 does not alter after ischemic stroke (Liu et al., 2008). Furthermore, age and genetic polymorphisms (e.g., cytokines, apolipoprotien E, Tau, and mitochondrial genes) affect BBB integrity (Song et al., 2004; Bennett et al., 2016). Therefore, studies concerning gender-, age-, and genetic-based difference in vasogenic edema formation in animal models and human patients are needed to elucidate the underlying mechanisms, the prediction of outcome and clinical management in various neurological diseases relevant to vasogenic edema.

## Conclusion

In conclusion, to the best of our knowledge, the present data show novel evidence that 67-kDa LR may be one of the up-stream regulators of dystrophin/p38 MAPK/VEGF/eNOS axis, which plays an important role in the maintenance of BBB integrity (Figure 6). Thus, our findings suggest that 67-kDa LR-mediated astroglio-vascular interface may be a therapeutic target for treatment of vasogenic edema.

## Data Availability

All datasets generated for this study are included in the manuscript and/or the Supplementary Files.

## Ethics Statement

All experimental protocols described below were approved by the Institutional Animal Care and Use Committee of Hallym University (Chuncheon, South Korea). Every effort was made to reduce the number of animals employed and to minimize animal’s discomfort.

## Author Contributions

T-CK designed and supervised the project, analyzed the data, and wrote the manuscript. All authors performed the experiments described in the manuscript.

## Conflict of Interest Statement

The authors declare that the research was conducted in the absence of any commercial or financial relationships that could be construed as a potential conflict of interest.
