# Supplementary material for: Dysfunction of 67-kDa Laminin Receptor Disrupts BBB Integrity via Impaired Dystrophin/AQP4 Complex and p38 MAPK/VEGF Activation Following Status Epilepticus
Source: Front Cell Neurosci. 2019 May 24;13:236. doi: 10.3389/fncel.2019.00236 (PMC6542995; doi:10.3389/fncel.2019.00236)
Supplement: Supplementary file 1 [file Data_Sheet_1.PDF]

Supplementary information

Dysfunction of 67-kDa laminin receptor disrupts BBB integrity via impaired dystrophin/AQP4 complex and p38 MAPK/VEGF activation following status epilepticus

Hana Park[1,2], Seo-Hyeon Choi[1,2], Min-Jeong Kong[1,2], Tae-Cheon, Kang[1,2*]

[1]Department of Anatomy and Neurobiology, College of Medicine, Hallym University, Chunchon 24252, South Korea

[2]Institute of Epilepsy Research, College of Medicine, Hallym University, Chunchon 24252, South Korea

Running title: Role of 67-kDa laminin receptor in vasogenic edema formation

* Correspondence to: T. -C. Kang, Department of Anatomy and Neurobiology, College of Medicine, Hallym University, Chunchon, Kangwon-Do 24252, South Korea; Tel: +82-33-248-2524; Fax: +82-33-248-2525; E-mail: tckang@hallym.ac.kr

Fig. 1

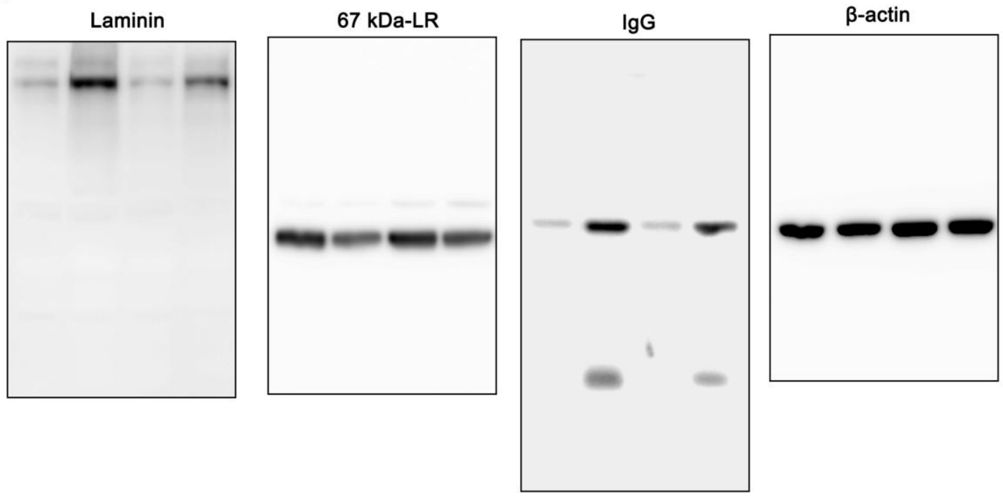

Fig. 3

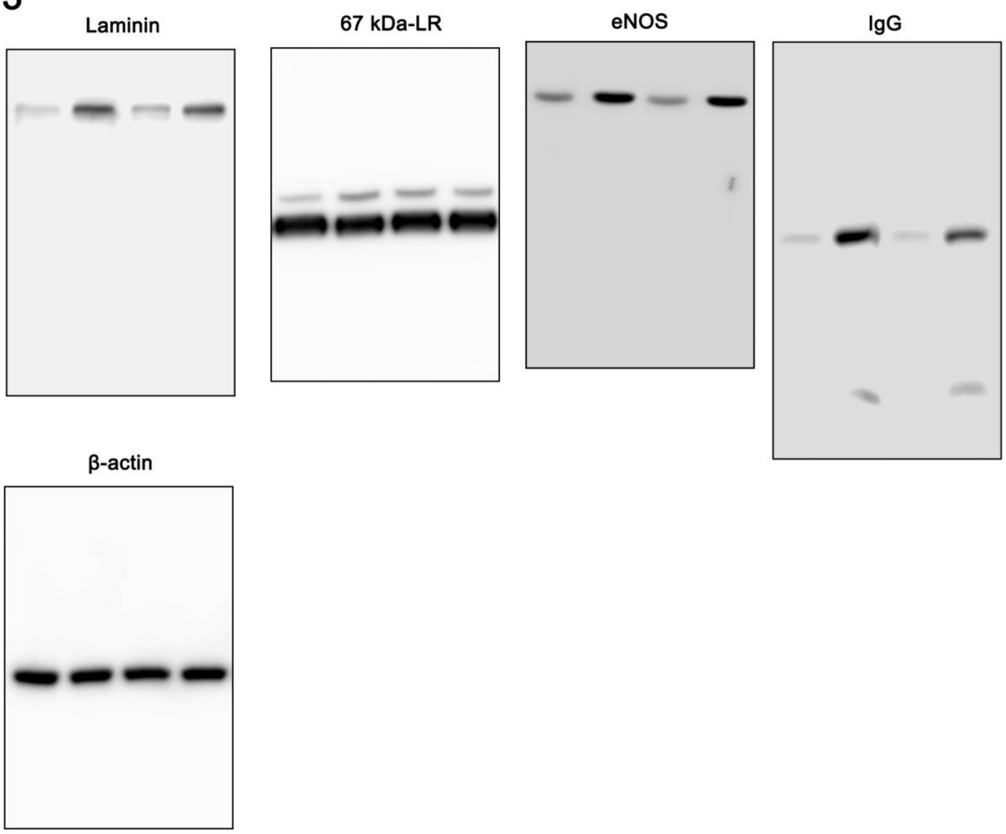

Supplementary Fig. 1. Full-length gel images of western blot data in Figs. 1 and 3.

Fig. 4

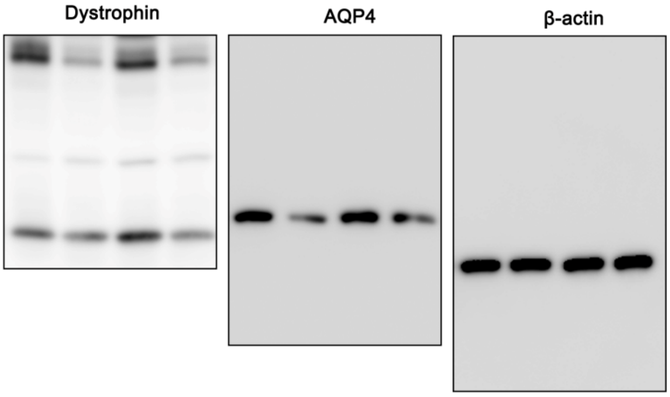

Fig. 5

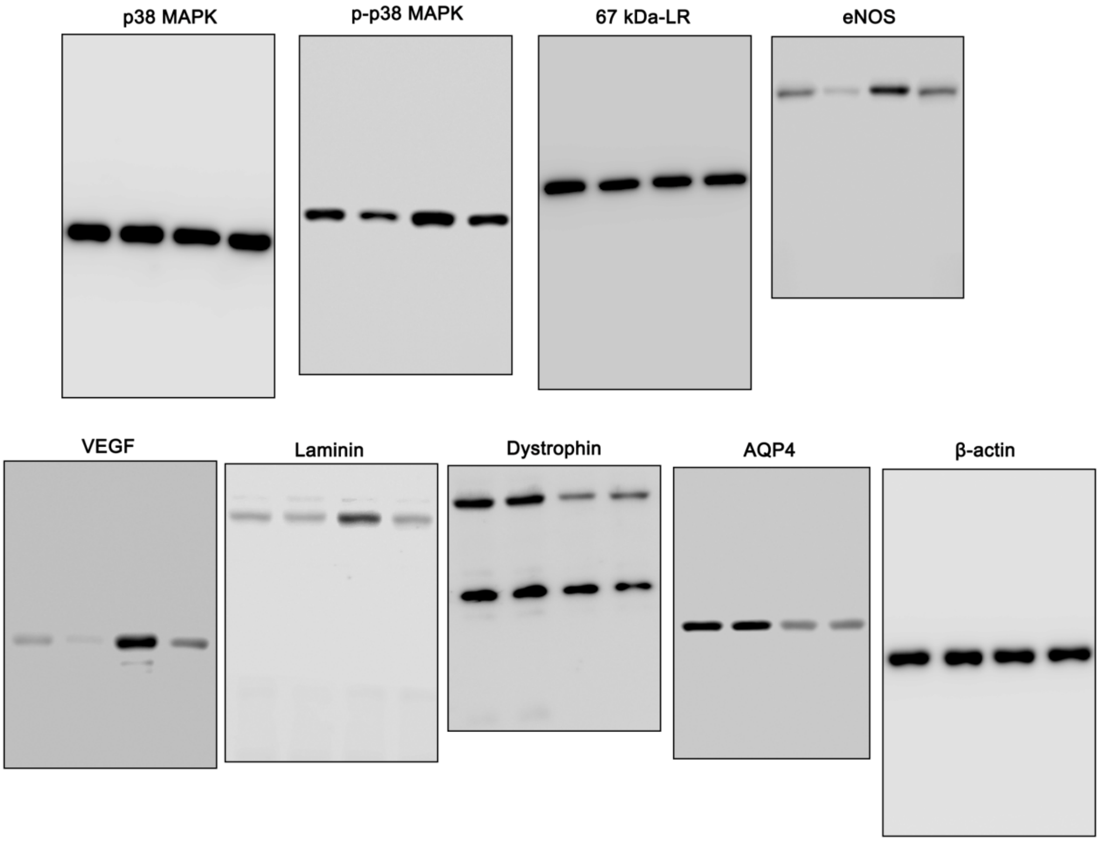

Supplementary Fig. 2. Full-length gel images of western blot data in Figs. 4 and 5.
